# Long intergenic non-protein coding RNA 511 promotes the progression of osteosarcoma cells through sponging microRNA 618 to upregulate the expression of maelstrom

**DOI:** 10.18632/aging.102109

**Published:** 2019-08-06

**Authors:** Wen Guo, Qing Yu, Ming Zhang, Fang Li, Yu Liu, Weiwei Jiang, Haitao Jiang, Haijun Li

**Affiliations:** 1 Department of Orthopaedic Surgery, Taizhou People’s Hospital, Jiangsu 225300, China; 2 Department of Neurology, Taizhou Hospital of Traditional Chinese Medicine, Jiangsu 225300, China

**Keywords:** osteosarcoma, LINC00511, miR-618, *MAEL*, aging, age-related diseases

## Abstract

Osteosarcoma is a tumor disease that commonly exists among young populations. Our research explored the role of the LINC00511/microRNA-618/*MAEL* axis in osteosarcoma.

Expression profiles of long non-coding RNAs (lncRNAs) in osteosarcoma (OS) tissues were constructed, and LINC00511 expression levels were verified with qRT-PCR. LncRNA-miRNA and miRNA-mRNA interactions were predicted. Validation was performed using a dual-luciferase reporter assay. Protein expression levels of *MAEL* were evaluated by Western blot assays. The effects of LINC00511, miR-618 and *MAEL* on the proliferation, viability, and metastasis of OS cells were detected using colony formation, cell counting kit-8 (CCK-8) and transwell assays, respectively. The apoptosis rates of OS cells were investigated using flow cytometry. The tumor-suppressing effect of LINC00511 silencing was also analyzed using a xenograft model in nude mice.

LINC00511 overexpression was observed in OS tissues and cell lines. Knockdown of LINC00511 in nude mice inhibited the tumorigenic ability of OS cells. Transfection-induced overexpression of LINC00511 and *MAEL*, as well as downregulation, highlighted the features of tumor cells, and LINC00511 overexpression reduced apoptosis *in vitro*.

LINC00511 was confirmed to be beneficial for osteosarcoma development via sponging miR-618 and increasing *MAEL* expression and may thus be considered a potential target for osteosarcoma therapy.

## INTRODUCTION

Osteosarcoma is a malignant primary bone tumor that commonly exists in young populations. Nevertheless, its molecular pathology is still unknown. The estimated incidence rate is 5 per million per year [1]. Patients without clinical signs of systematic spread show 5-year survival rates of 60–80%, whereas patients with metastasis at diagnosis exhibit 5-year survival rates of 20–30% [2]. Recently, emerging evidence has demonstrated that microRNA dysregulation may be a useful biomarker for osteosarcoma diagnosis and treatment. MicroRNAs (miRNAs) are endogenous small (~22 nucleotides) non-coding RNAs functioning at the post-transcriptional level that regulate the expression of target genes [3, 4]. MiRNAs are involved in several complicated cellular physiological processes [2]. Importantly, several miRNAs have been found to be involved in osteosarcoma, such as miR-1270 [5], miR-222 [6], miR-299-5p [5], and miR-16 [7]. MiR-618 deregulation has previously been linked to a number of malignancies, including hepatocellular carcinoma, male breast cancer, and Barrett’s esophageal cancer, hinting at the potential of this miRNA as a cancer biomarker and therapeutic target [8]. Despite numerous studies, research on the interaction of miR-618 with cancer, however, and the concrete mechanism through which miR-618 exerts its role in OS, especially its interaction with lncRNAs, remains obscure.

At the same time, some researchers found dysregulated expression profiles of other non-coding RNAs, such as long non-coding RNAs, in osteosarcoma tissues [9]. Long non-coding RNAs are also known as lncRNAs and are non-protein-coding transcripts that consist of over 200 nucleotides. Many scientists have reported that lncRNAs may greatly influence the initiation as well as the development of many human diseases [10]; these lncRNAs include lncRNA ZEB1-AS1 [11], MALAT1 [12], and XIST [5]. However, it is still not clear whether malignant osteosarcoma can be induced by other lncRNAs, especially oncogene long intergenic noncoding RNA 00511 (LINC00511), which has been noted to play a role in pancreatic ductal adenocarcinoma [13] and non-small-lung cancer [14] through sponging miRNAs and modulating the expression of downstream mRNA. To explore the role of LINC00511 in osteosarcoma, we measured its levels in OS cells, detected variations in cell biological functions and investigated the underlying molecular pathways.

The maelstrom (*MAEL*) gene is a cancer-testis gene that is expressed in only spermatocytes [5]. However, in breast cancer cells, *MAEL* is activated by demethylation [15]. In addition to breast cancer, increasing evidence has shown that abnormal expression of *MAEL* exists in other human cancers, including colon, liver, and bladder cancer [16]. However, questions about what role *MAEL* plays in osteosarcoma are still largely known.

Here, expression profiles of lncRNAs in osteosarcoma were constructed, and activated LINC00511 was selected as the research focus. Then, *in vivo* and *in vitro* experiments were conducted to investigate the in-depth mechanism of abnormal LINC00511 expression in OS development and determine the impact of lncRNAs on OS. Overall, we discovered that LINC00511 upregulation was beneficial for *MAEL* expression. Furthermore, sponging and downregulating the expression of miR-618 enhanced the tumorigenic and metastatic abilities of OS cells.

## RESULTS

### Expression of LINC00511 in osteosarcoma tissues and cells

**Figure 1:**
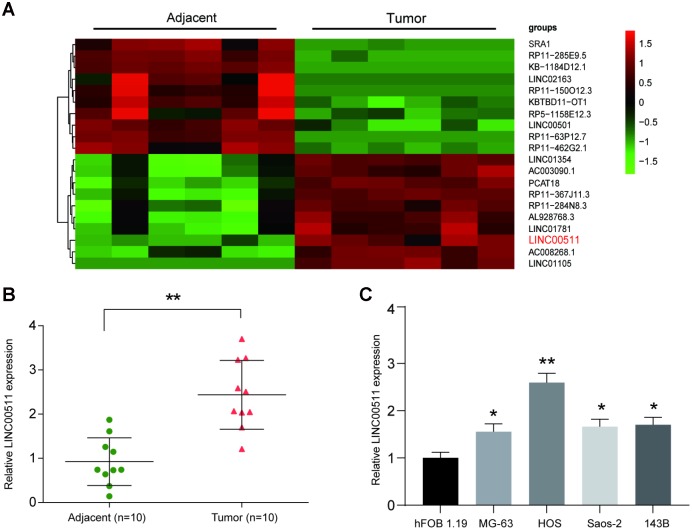
**LncRNA microarray analysis and high expression of LINC00511 in OS.** (**A**) Heatmap of lncRNA expression in OS tissues (n=6). LncRNAs were selected based on Flod change value>2 and *P*<0.05; the overexpressed lncRNAs are shown in red, and the downregulated lncRNAs are shown in green. (**B**) The expression of LINC00511 in Tumor tissues was higher than that in adjacent tissues as detected by qRT-PCR. **P<0.01 compared to the Adjacent group. (**C**) The expression of LINC00511 in OS cell lines, including MG-63, HOS, Saos-2 and 143B. *P<0.05 and **P<0.01 compared to the noncancerous osteoblast cell line hFOB 1.19.

Microarray analysis showed that the expression profile of lncRNAs was significantly changed in OS tissues; lncRNAs with a fold change-value >2 and *P*<0.05 are shown in Figure 1A. Of these, LINC00511 showed the largest increase in tumors compared to its expression in adjacent tissues. We next evaluated the LINC00511 expression level in 10 OS patient samples and compared them with the levels in adjacent tissues. The results showed that the LINC00511 expression level was higher in tumor tissues than in adjacent tissues (Figure 1B, *P*<0.01). Then, a qRT-PCR assay was performed to determine the expression of LINC00511 in various OS cell lines (MG-63, HOS, Saos-2 and 143B); as shown in Figure 1C, LINC00511 was higher in all OS cell lines than in hFOB 1.19 osteoblasts and reached its highest level in HOS cells. As a result, LINC00511 overexpression was observed in OS tissues and cells.

### Effects of LINC00511 on OS cell proliferation, apoptosis, migration and invasion *in vitro*

**Figure 2:**
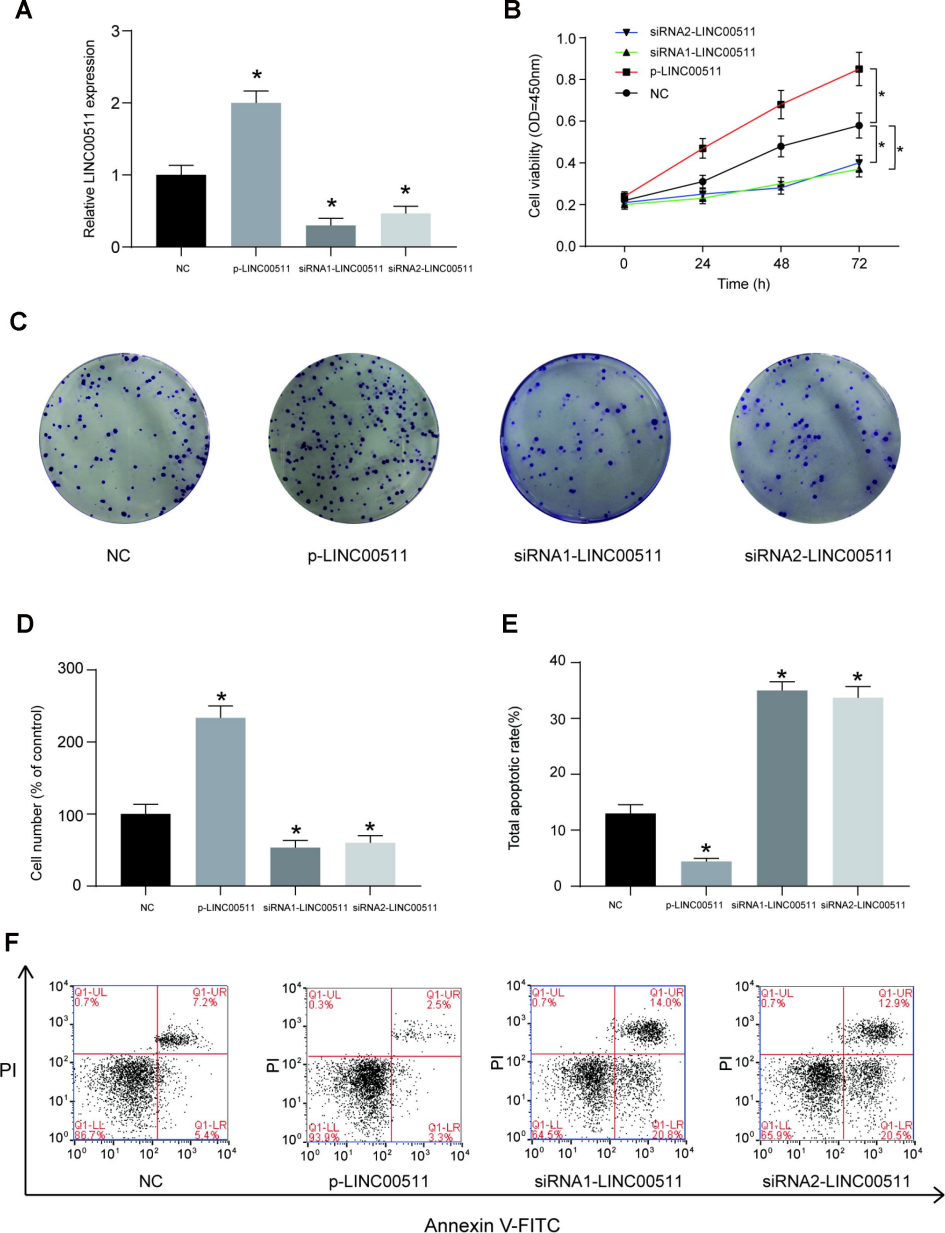
**LINC00511 mediates the cell proliferation and apoptosis of HOS cells *in vitro.*** (**A**) The relative expression level of LINC00511 was changed in HOS cells transfected with p-LINC00511- or LINC00511-targeting siRNA. P-LINC00511 is a LINC00511 overexpression vector, and its transfection induced a high level of LINC00511 expression. In contrast, transfection of specific LINC00511-targeting siRNA caused low expression levels of LINC00511 in HOS cells. (**B**) CCK-8 assays showed that the proliferation ability is changed in HOS cells with LINC00511 dysregulation. Up- or downregulation of LINC00511 was induced by pre-transfection with p-LINC00511 or specific siRNA. (**C–D**) Colony formation ability of HOS cells with altered expression of LINC00511. (**E–F**) Apoptosis rate of HOS cells with altered expression of LINC00511. *P < 0.05 compared to the NC group.

**Figure 3:**
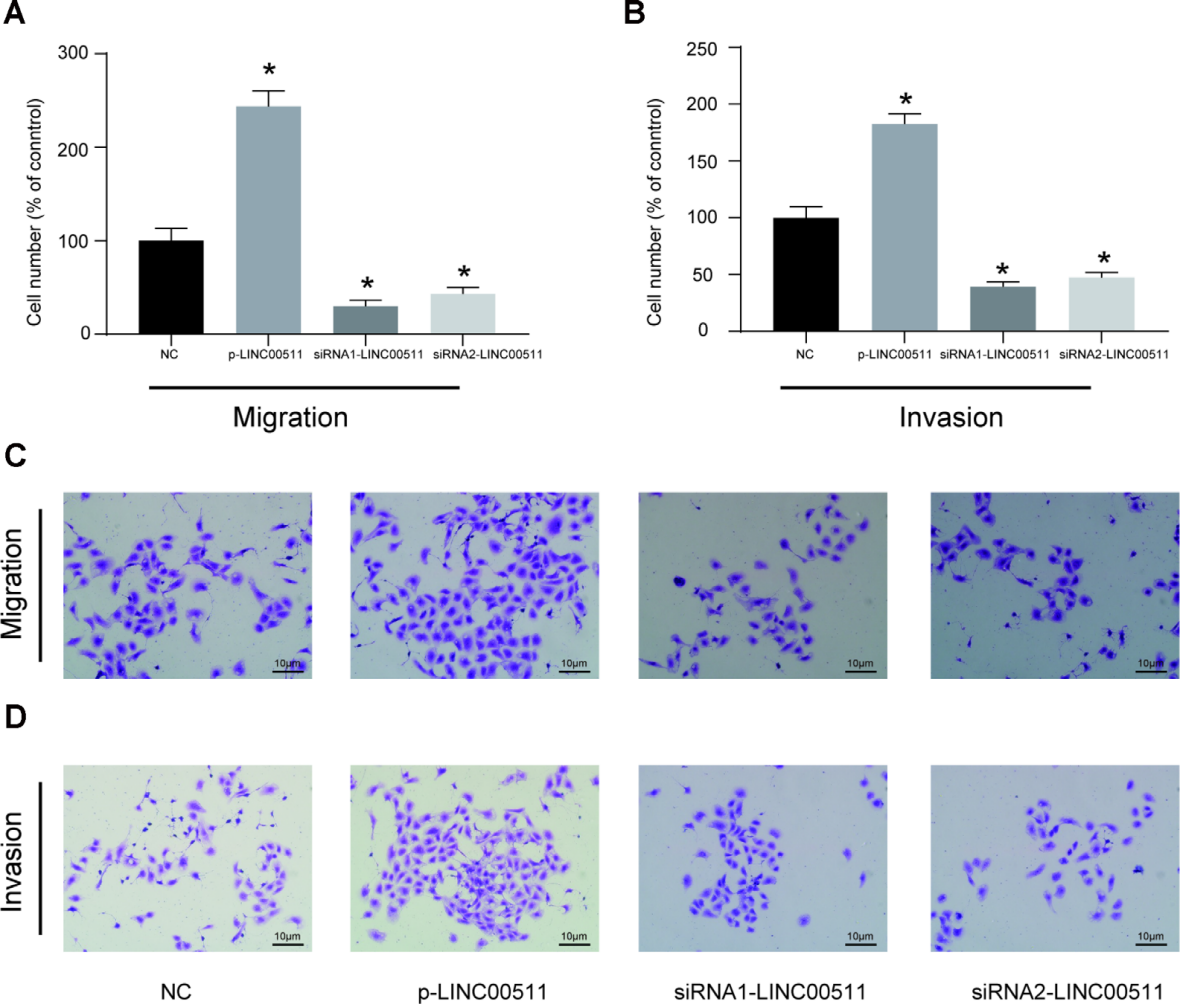
***In vitro* migration and invasion activities of HOS cells with LINC00511 dysregulation.** (**A**, **C**) *In vitro* migration ability of HOS cells with dysregulation of LINC00511. (**B**, **D**) *In vitro* invasion ability of HOS cells with dysregulation of LINC00511. Both migration and invasion abilities were detected using a transwell assay. LINC00511 dysregulation in HOS cells was induced by the pre-transfection of p-LINC00511 (upregulation) or specific siRNA (downregulation). *P < 0.05 compared to the NC group.

The HOS cell line, which had the highest expression of LINC00511, was selected for further cell-based experiments. First, HOS cells were divided into NC (cells transfected with empty pcDNA3.1 plasmid), p-LINC00511 (cells transfected with pcDNA3.1-LINC00511), siRNA1 (cells transfected with LINC00511-targeting siRNA1) and siRNA2 (cells transfected with LINC00511-targeting siRNA2) groups. Compared to that in the NC group, expression of LINC00511 in the p-LINC00511 group was significantly increased, whereas expression in the siRNA-LINC0051 groups was decreased (Figure 2A, *P*<0.05). Both the CCK-8 (Figure 2B) and colony formation assays (Figure 2C, 2D) showed that compared to that in empty plasmid-transfected HOS cells, proliferation ability was significantly decreased in HOS cells with siRNA-induced LINC00511 silencing but was enhanced in cells with LINC00511 overexpression (*P*<0.05). On the other hand, LINC00511 silencing observably increased the apoptosis rate of HOS cells, but for cells with LINC00511 overexpression, the apoptosis rate was reduced (Figure 2E, 2F, *P*<0.05). Furthermore, Transwell assays showed that silencing LINC00511 significantly inhibited the migration (Figure 3A, 3C) and invasion (Figure 3B, 3D) of HOS cells *in vitro*, whereas migration and invasion were increased in HOS cells overexpressing LINC00511 (*P*<0.05).

### LINC00511 silencing inhibits the growth of xenografts formed by HOS cells in nude mice

**Figure 4:**
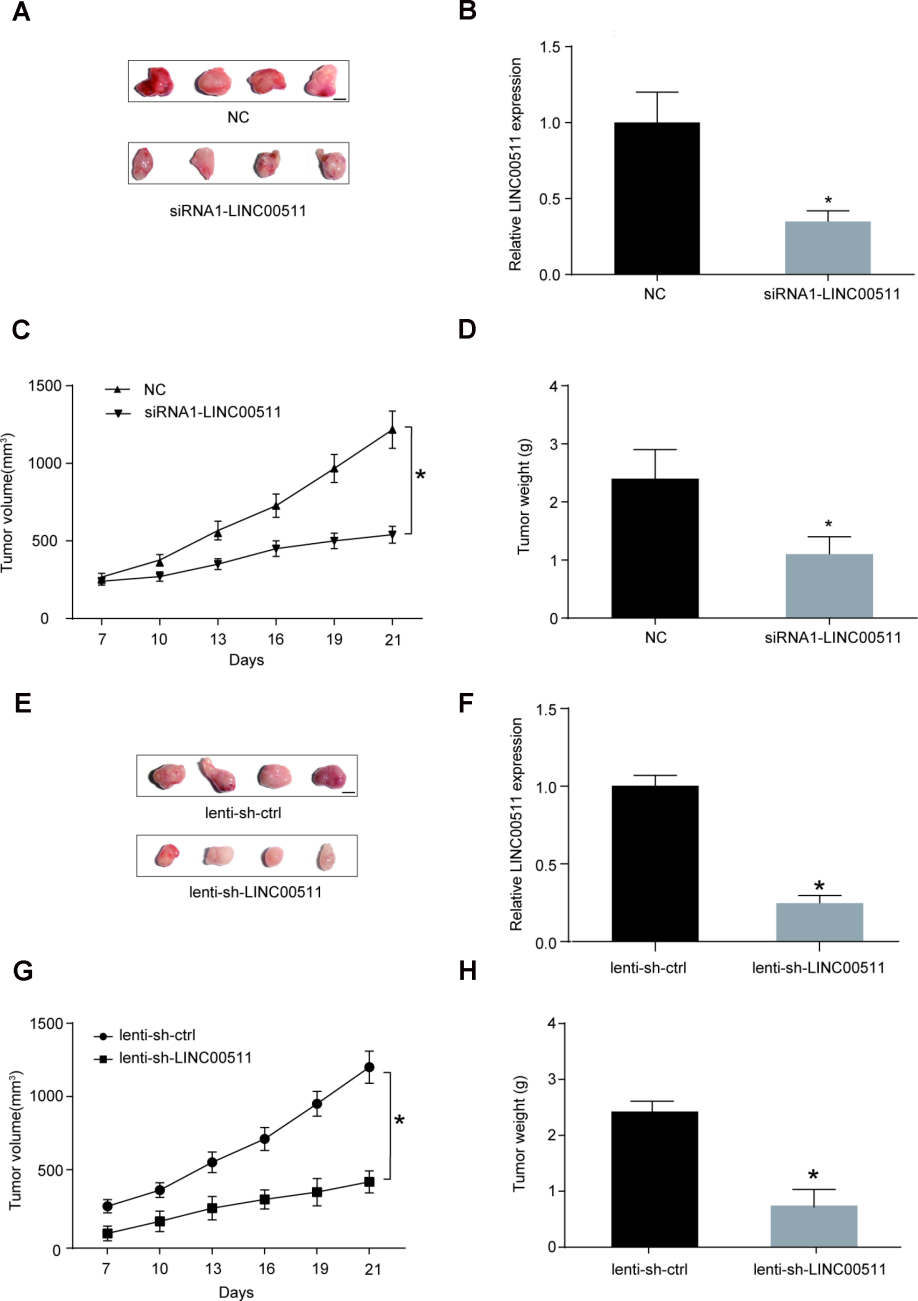
**LINC00511 promotes the tumorigenesis of OS in nude mice.** (**A**) Xenografts formed by HOS cells with normal (upper) or downregulated (bottom) expression of LINC00511. Tumor tissues were harvested after 3 weeks of implantation (Scale bar, 0.5 cm). (**B**) Expression level of LINC00511 in the xenografts tissues. *P<0.05 compared with NC group. (**C–D**) SiRNA-induced downregulation of LINC00511 inhibited the growth of xenografts in nude mice. The volumes of xenografts were measured every 3 days, and their weights were measured after harvesting. (**E**) Xenografts formed by HOS cells with normal (upper) or downregulated (bottom) expression of LINC00511. Tumor tissues were harvested after 3 weeks of implantation (Scale bar, 0.5 cm). Lenti-sh-LINC00511 cells were transfected with lentivirus-mediated shRNA targeting LINC00511. Lenti-sh-ctrl means cells were transfected with shRNA lentiviral particles with nontargeting scrambled shRNA sequences. (**F**) The LINC00511 expression level in lenti-sh-LINC00511 group was markedly decreased compared to that in the lenti-sh-ctrl group. (**G**–**H**) ShRNA-induced downregulation of LINC00511 inhibited the growth of xenografts in nude mice. The volumes of xenografts were measured every 3 days, and their weights were measured after harvesting. *P<0.05 compared to the lenti-sh-ctrl group.

The ability of LINC00511 downregulation to inhibit the growth and tumorigenesis of OS *in vivo* was evaluated in a xenograft nude mouse model. HOS cells pre-transfected with siRNA-LINC00511 or siRNA-control and lentivirus-mediated shRNA-LINC00511 or shRNA-control were injected subcutaneously into the backs of nude mice respectively. At the end of the experiment (the 21^st^ day), the mice were euthanized, and tumor tissues were excised; the sizes (Figure 4A, 4E) and weights (Figure 4D, 4H, *P*<0.05) of xenografts formed by HOS cells with low LINC00511 expression were clearly lower than those formed by normal HOS cells. The LINC00511 expression level in the lenti-sh-LINC00511 and siRNA-LINC00511 group were markedly decreased compared to that in the lenti-sh-ctrl group or siRNA-control group respectively (Figure 4B, 4F, *P*<0.05). During the period of xenograft growth in nude mice, the measurement of tumor volumes was performed every three days. The results showed that the volumes of xenografts formed by HOS cells with low LINC00511 expression were significantly less than those formed by normal HOS cells (Figure 4C, 4G).

### Target relationship between LINC00511 and miR-618

**Figure 5:**
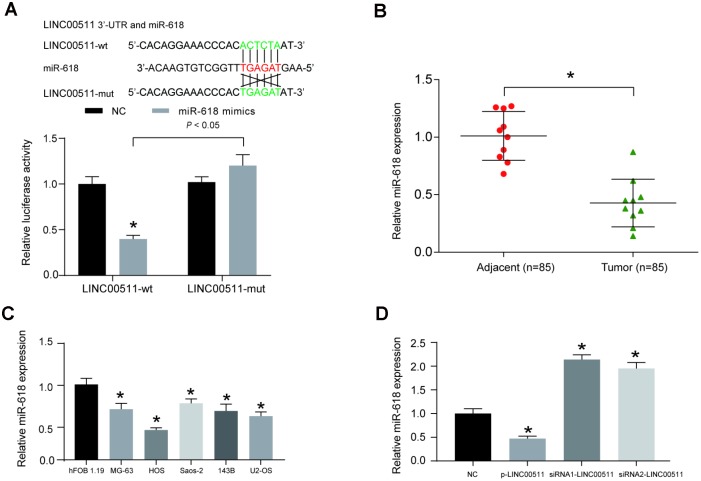
**LINC00511 suppressed the expression of miR-618 in HOS cells.** (**A**) The target relationship between miR-618 and LINC00511 was predicted by bioinformatics analysis (upper) and validated by dual-luciferase reporter assays (bottom). MiR-618 mimics significantly inhibit the fluorescence activity of the reporter vector carrying wild-type LINC00511, but not mutant LINC00511. *P<0.05 compared with the NC group. (**B**) The expression of miR-618 in OS tissues and adjacent tissues was measured using qRT-PCR. *P<0.05 compared to the adjacent group. (**C**) Downregulated expression of miR-618 in OS cell lines detected by qRT-PCR analysis. *P<0.05 compared with the noncancerous osteoblast cell line hFOB 1.19. (**D**) Expression of miR-618 in HOS cells is inhibited by LINC00511. *P<0.05 compared to the NC group.

Bioinformatics analysis based on the miRcode v11 database was performed to predict the target miRNA of LINC00511; miR-618 presented the highest combination potential with LINC00511. The putative binding site between miR-618 and LINC00511 is shown in Figure 5A. Then, a dual-luciferase reporter assay was used to validate the targeted correlation between LINC00511 and miR-618 *in vitro*; the results demonstrated that miR-618 mimics were capable of significantly inhibiting the 3′-UTR activity of the LINC00511 luciferase reporter containing the binding site of miR-618 (*P*<0.05). This inhibition was completely abolished when the binding site was mutated (Figure 5A). Meanwhile, the expression levels of other miRNAs with a targeted relationship to LINC00511 were detected. Nine miRNAs expression levels were detected in OS tissues and adjacent normal tissues by qRT-PCR assay. The miR-618 expression level in OS tumor tissues was lowest among the 9 miRNAs (Supplementary Figure 1). MiR-618 was selected as the research object. The miR-618 expression level was significantly lower in 10 OS patient samples than in adjacent normal samples (Figure 5B, *P*<0.05). The expression of miR-618 in OS cell lines (including MG-63, HOS, Saos-2, 143B and U2-OS) was significantly lower than that in the non-cancerous hFOB 1.19 cell line (Figure 5C, *P*<0.05). Transfection of p-LINC00511 (a LINC00511 overexpression vector) significantly suppressed the expression of miR-618 in HOS cells, but siRNA-LINC00511 transfection increased the miR-618 expression level greatly compared to that in normal cells (Figure 5D, *P*<0.01).

### MiR-618 suppresses the *in vitro* activity of OS cells

**Figure 6:**
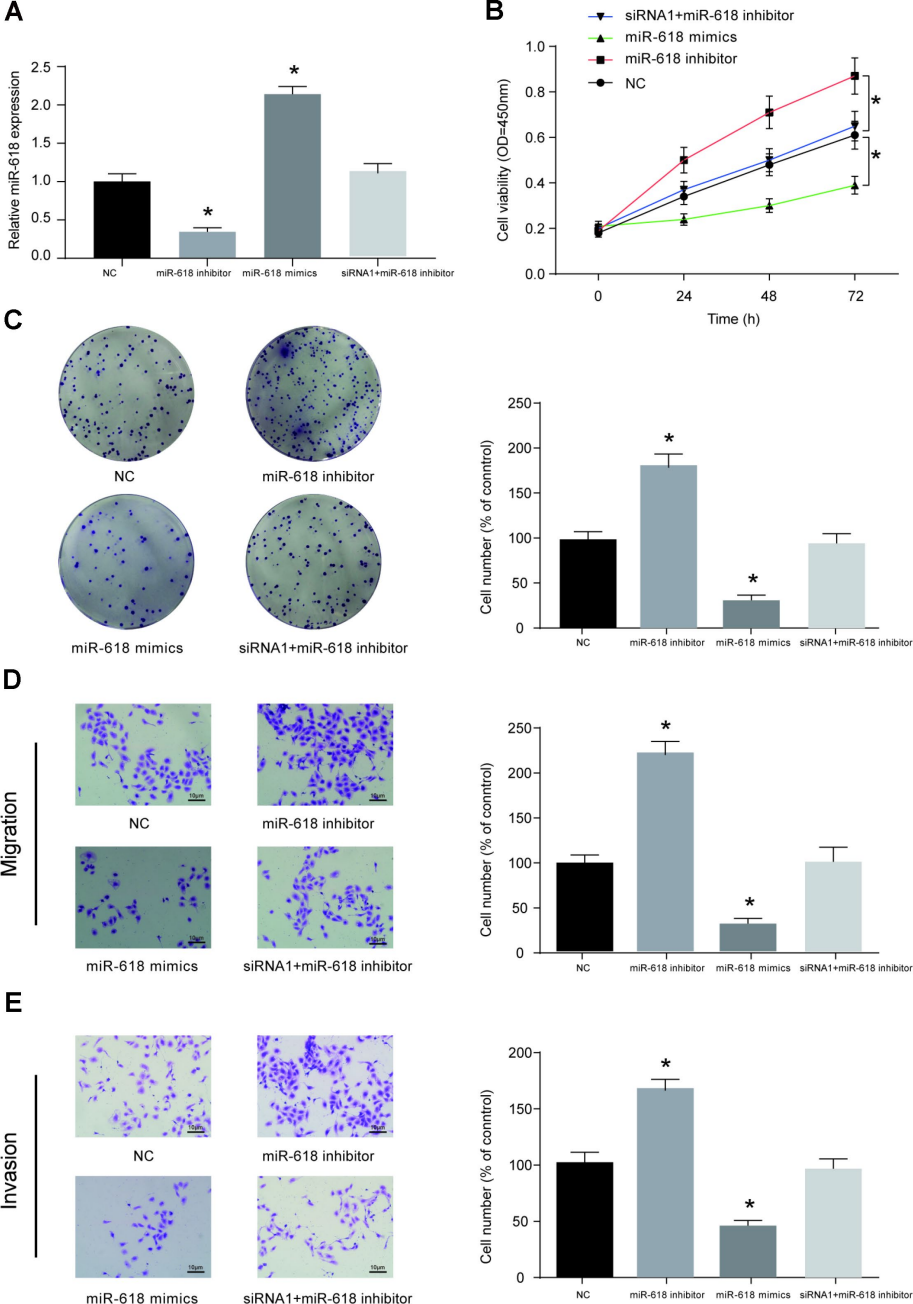
**MiR-618 inhibits the *in vitro* activity of OS cells.** (**A**) Expression profiles of miR-618 in HOS cells transfected with miR-618 mimics (upregulation), miR-618 inhibitor (downregulation) or si-LINC00511+miR-618 inhibitor (not changed). (**B–E**) CCK-8, colony formation and transwell assays of the proliferation, migration and invasion ability of HOS cells with miR-618 dysregulation. (**B**) CCK-8 assay for cell viability. (**C**) Colony formation assay for cell proliferation. (**D**) Transwell assay for cell migration. (**E**) Transwell assay for cell invasion. *P<0.05 compared to the NC group.

HOS cells were pre-transfected with miR-NC, miR-618 inhibitor, miR-618 mimics or siRNA1-LINC00511+miR-618 inhibitor. Then, the miR-618 expression level in each group was measured using qRT-PCR, and the results showed that miR-618 mimics could significantly promote the expression of miR-618, while the miR-618 inhibitor decreased the expression of miR-618 in HOS cells. Co-transfection of siRNA-LINC00511 and miR-618 inhibitor did not alter the expression of miR-618 (Figure 6A). CCK-8 (Figure 6B) and colony formation (Figure 6C) assays showed that compared to that in miR-NC transfected cells, proliferation ability was significantly enhanced in HOS cells transfected with the miR-618 inhibitor but was reduced in cells transfected with miR-618 mimics (*P*<0.01). Additionally, a transwell assay showed that miR-618 mimics could induce less HOS cell migration and cell invasion *in vitro*. In contrast, a completely opposite outcome was obtained from the miR-618 inhibitor transwell assay (Figure 6D, 6E, *P*<0.01).

### Expression of *MAEL* in OS is mediated by the upstream miR-618 and LINC00511

**Figure 7:**
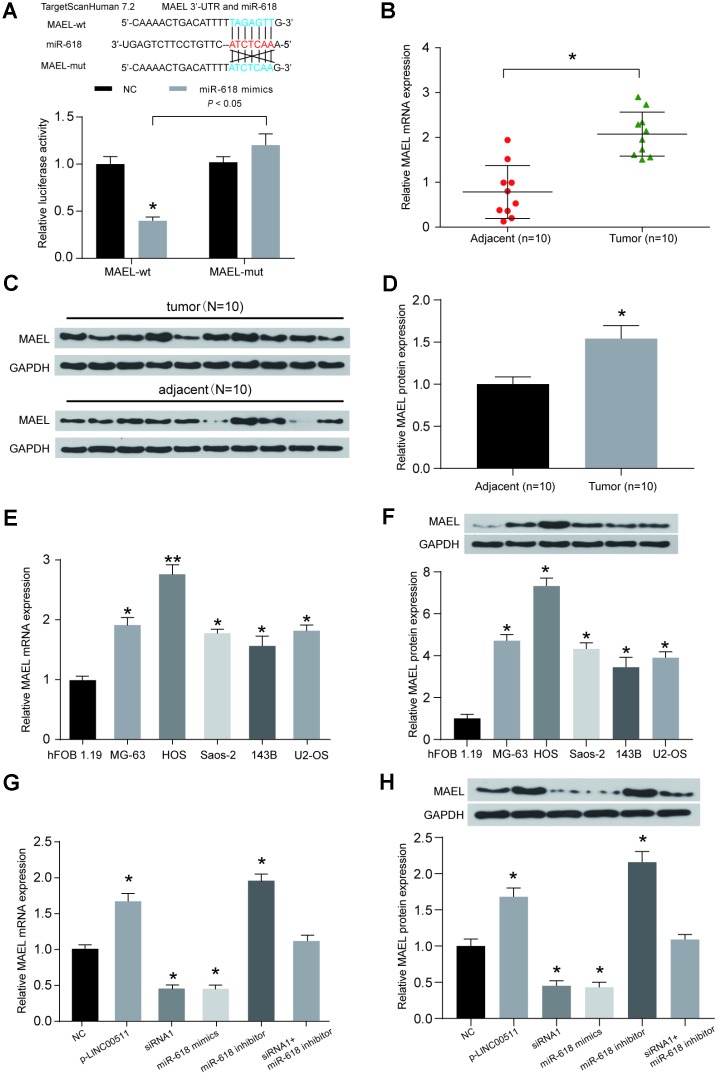
**The expression of *MAEL* is mediated by both miR-618 and LINC00511.** (**A**) Binding sites between miR-618 and *MAEL* were predicted by TargetScanHuman7.2. Then, the target relationship between miR-618 and *MAEL* was validated by a dual-luciferase reporter assay. MiR-618 mimics significantly inhibited the expression of luciferase reporter vectors carrying the wild-type *MAEL* sequence but not the mutated *MAEL* sequence. *P<0.05 compared to the NC group. (**B**) A qRT-PCR assay shows that *MAEL* mRNA expression was higher in OS tissues than in corresponding normal tissues. *P<0.05 compared to the adjacent group. (**C–D**) *MAEL* protein expression levels were detected in OS tissues and adjacent normal tissues by Western blot assay (N=10). (**E–F**) *MAEL* mRNA (**E**) and protein (**F**) expression levels in OS cell lines. *P<0.05 and **P<0.01 compared to the noncancerous osteoblast cell line hFOB 1.19. (**G–H**) *MAEL* mRNA (**G**) and protein (**H**) expression levels in HOS cells transfected with NC, p-LINC00511, siRNA-LINC00511, and miR-618 inhibitor or mimics were tested by qRT-PCR and Western blot assays. *P<0.05 compared to the NC group.

The downstream mRNA target of miR-618 was predicted by the TargetScan v7.2 database; *MAEL* was selected as a candidate. The binding site between miR-618 and *MAEL* is shown in Figure 7A. A dual-luciferase reporter assay showed that miR-618 mimics could target the predicted binding sites in the 3′-UTR of *MAEL* and then inhibit its expression (Figure 7A). High *MAEL* mRNA expression was confirmed in OS tissues compared with that in adjacent normal tissues (Figure 7B, *P*<0.05). Additionally, the protein expression of *MAEL* was higher in OS tissues than in normal tissues by Western blot assay (Figure 7C, 7D, *P*<0.05, N=10). The mRNA and protein expression of *MAEL* in OS cell lines (including MG-63, HOS, Saos-2, 143B and U2-OS) was significantly higher than that in the non-cancerous hFOB 1.19 cell line (Figure 7E, 7F, *P*<0.05). Subsequent cell-based studies showed that transfection with p-LINC00511 or the miR-618 inhibitor significantly increased the mRNA and protein expression of *MAEL* in HOS cells, but both siRNA-LINC00511 and miR-618 mimic transfection reduced *MAEL* expression greatly compared to that in normal HOS cells (Figure 7G, 7H, *P*<0.01).

### Downregulation of *MAEL* suppresses the activity of OS cells *in vitro*

**Figure 8:**
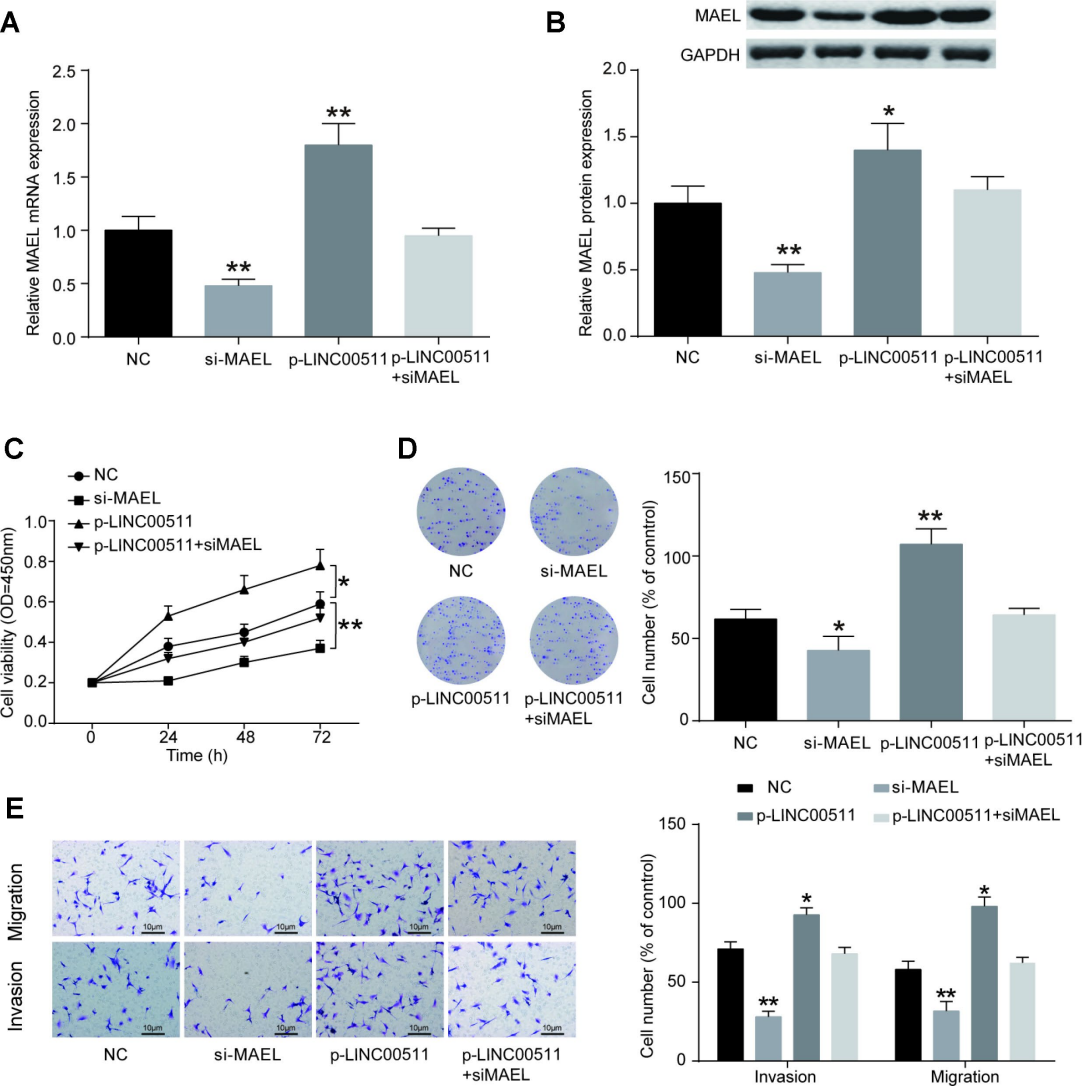
**MiR-618 promotes the tumor activity of OS cells *in vitro*.** (**A**) Specific siRNA inhibited the expression of *MAEL* mRNA in HOS cells. (**B**) *MAEL* protein expression was dramatically lower in the si-MEAL group than in the NC group. (**C–E**) CCK-8, colony formation and transwell assays of the proliferation, migration and invasion abilities of HOS cells with *MAEL* silencing. (**C**) CCK-8 assay for cell viability. (**D**) Colony formation assay for cell proliferation. (**E**) Transwell assays for cell migration and invasion. *P<0.05 and **P<0.01 compared to the NC group.

Whether the normal expression of *MAEL* is essential for the growth and tumorigenesis of OS was investigated *in vitro*. *MAEL* mRNA expression levels in cells were measured with qRT-PCR. Initially, HOS cells were pre-transfected with siRNA-*MAEL,* p-LINC00511 or siRNA-NC. We then observed that the *MAEL* mRNA (Figure 8A, *P*<0.01) and protein (Figure 8B, *P*<0.01) expression levels were significantly decreased by siRNA-*MAEL*. CCK-8 (Figure 8C) and colony formation (Figure 8D) assays showed that compared with that in siRNA-NC-transfected HOS cells, proliferation ability was significantly reduced in HOS cells transfected with siRNA-*MAEL* (*P*<0.01). Furthermore, Transwell assays also showed that siRNA-*MAEL* transfection could decrease the migration and invasion of HOS cells *in vitro* (Figure 8E, *P*<0.01). In contrast, a completely opposite outcome was obtained from the p-LINC00511 group.

## DISCUSSION

In this study, we observed significantly higher LINC00511 expression in OS tissues than in adjacent healthy tissues. Moreover, siRNA-induced LINC00511 silencing greatly suppressed OS cell tumorigenic and metastatic activities. These encouraging results indicated that LINC00511 plays an oncogenic role in initiating and developing OS. Similarly, high expression of LINC00511 has been reported in several cancers, such as tongue squamous cell carcinoma [5] pancreatic ductal adenocarcinoma [13], and non-small-cell lung cancer [14]. Cancer cells overexpressing LINC00511 showed enhanced proliferation, tumorigenesis, metastasis and invasiveness. In addition to LINC00511, several lncRNAs have also been shown to be dysregulated and affect the development of OS [16–18]. For example, lncRNA miR210HG was increased in OS tissues, and the aberrantly enhanced expression of miR210HG predicted a poor prognosis as well as a lower survival rate in OS patients [17]. Additionally, reduced expression of some lncRNAs is also considered an indispensable part of OS development; for example, overexpression of lncRNA GAS5 will greatly suppress the proliferation, migration and EMT (epithelial-mesenchymal transition) of OS cells [17]. In our study, we established an OS-associated lncRNA expression profile; moreover, the results showed that most lncRNAs were significantly up- or downregulated in OS patients’ tumor tissues. The tumor-promoting role and potential mechanism of LINC00511 in OS were uncovered. However, the tumor-associated role of other dysregulated lncRNAs in OS is thus far unknown. Consequently, more and better additional studies are required. Recent studies have shown that lncRNAs could act as an endogenous miRNA “sponge” by interacting with miRNAs to mediate the expression of mRNA at the post-transcriptional level [19, 20]. For example, Zhao et al. [13] found that LINC00511 increased the expression of VEGFA through sponging miR-29b-3p, subsequently promoting cell proliferation, migration and endothelial tube formation in pancreatic ductal adenocarcinoma. Ding et al. [6] also discovered that LINC00511 was capable of enhancing the proliferative ability and invasive ability of tongue squamous cell carcinoma cells by sponging miR-765 to facilitate LAMC2 expression. Additionally, lncRNAs are able to modulate the expression of target genes by directly interacting with RNA binding proteins [19]. Sun et al. [14] showed that LINC00511 can bind to the histone methyltransferase enhancer of zeste homolog 2 (EZH2) and then suppress the expression of p57 to promote the initiation and development of non-small-cell lung cancer. In our experiments, we identified miR-618 as a potential target for LINC00511 using sequence complementarity analysis and dual-luciferase reporter assays. An obvious inverse correlation between miR-618 and LINC00511 in HOS cells was observed. The tumor-suppressing role of miR-618 has been reported in several cancer types, such as prostate cancer [21] and thyroid carcinoma [22, 23]. However, to the best of our knowledge, the role of miR-618 in OS remains largely unknown. Here, we found that the *in vitro* activities of growth, metastasis, and invasion in OS cells transfected with miR-618 mimics were weakened, but those in OS cells transfected with the miR-618 inhibitor were enhanced. When the cells were co-transfected with LINC00511-targeting siRNA and miR-618 inhibitor, their proliferation, migration, and invasion were not obviously different from those of cells transfected with target-free siRNA or mimics. These results showed that the tumor-promoting role of LINC00511 in OS may largely depend on the restriction on the activity of miR-618. However, as mentioned above, several other miRNAs can be mediated by LINC00511 and then affect the initiation or development of diseases [6, 13]. Therefore, we cannot refute the possibility that high expression of LINC00511 can promote the development of OS by mediating the expression of miRNAs (i.e., miR-29b-3p and miR-765) other than miR-618. Finally, *MAEL* was identified as an immediate target of miR-618. The expression level of *MAEL* was negatively mediated by miR-618 in OS cells, and downregulation of *MAEL* plays a tumor-inhibiting role in OS cells. As a cancer-testis (or cancer-germline) gene, *MAEL* is predominantly expressed in germline cells and is essential for meiosis and spermatogenesis under normal conditions [24, 25]. However, abnormal expression of *MAEL* has also been identified in multiple human cancer types [26, 27]. For example, Liu et al. [28] reported that silencing *MAEL* could effectively prevent hepatoma carcinoma cell growth and migration *in vitro* and xenograft formation in nude mice. Li et al. [29] suggested that overexpression of *MAEL*, caused by gene amplification and/or decreased miR-618, has a critical oncogenic role in the pathogenesis of bladder urothelial carcinoma by downregulating MTSS1 at the epigenetic level. Wu et al. [18] proposed that the *MAEL* gene and other lncRNAs might also participate in breast tumorigenesis. Like their observations, we found increased expression of *MAEL* in OS cells. Furthermore, silencing *MAEL* by specific siRNA transfection caused the proliferative, migrative and invasive abilities of OS cells to be significantly inhibited. Our results indicate that *MAEL* contributes to OS tumorigenesis and metastasis, but further study is still necessary to validate the concrete mechanisms.

We identified that reduced LINC00511 expression was enough to inhibit the proliferative ability and tumorigenesis of OS cells both *in vitro* and *in vivo*. The tumor-promoting role of LINC00511 in OS is produced mainly by sponging miR-618 to facilitate the expression of *MAEL*. Our research provided three promising biomarkers as well as targets for further investigation regarding the diagnosis or therapeutic intervention of OS.

## METHODS

### Patients and tissue samples

OS tissues and associated adjacent non-tumorous tissues were obtained from 10 patients who had surgical resection at the Bone Tumor Center, Taizhou People’s Hospital, between March 2015 and September 2017. OS patients who participated in this study had not previously received chemotherapy or radiotherapy. All tissue specimens were frozen in liquid nitrogen immediately and stored at −80°C until use. An agreement was reached with the Institutional Ethics Committee of Taizhou People’s Hospital; moreover, approval was obtained from all patients before actions were taken.

### Cell lines and culture

The human osteoblast cell line hFOB 1.19 and the human osteosarcoma (OS) cell lines MG-63, HOS, Saos-2, and 143B were obtained from the American Type Culture Collection (ATCC). hFOB 1.19 cells were incubated in DMEM/F-12 medium supplemented with 150 mg/L L-glutamine, 1.5 g/L NaHCO_3_ and 10% fetal bovine serum (FBS). MG-63 and 143B cells were cultured in high glucose DMEM supplemented with 10% FBS. HOS cells were cultured in MEM-EBSS medium supplemented with 10% FBS. Saos-2 cells were cultured in McCoy’s 5A medium supplemented with 2.2 g/L NaHCO_3_ and 15% FBS. Human osteosarcoma cell lines were incubated at 37°C in a humidified atmosphere with 5% CO_2_. The incubation environment of the human osteoblast cell line was 34°C with 5% CO_2_.

### RNA extraction and qRT-PCR

RNA extraction was conducted using TRIzol™ (Invitrogen, Carlsbad, CA, USA) according to the manufacturer’s instructions. A NanoDrop-1000 (Thermo Fisher Scientific, Waltham, MA, USA) was adopted to test the concentrations and for quality control purposes. A complementary DNA library of all samples was constructed from the extracted RNA samples (5 μg) using a SuperScript^®^ IV First-Strand Synthesis System (Invitrogen) in accordance with the recommended protocol. Subsequently, real-time PCR was performed using Arrystar SYBR^®^ Green Real-time qPCR Master Mix (Arraystar, Rockville, MD, USA). MiRNA was normalized to U6, and lncRNA and mRNA were normalized to GAPDH. The sequences of the primers are presented in Table 1. Finally, the data were processed using the 2^−ΔΔCt^ relative expression method.

**Table 1:** Primer sequences for qRT-PCR.

**Primer**	**Accession number**	**Sequence (5′-3′)**
LINC00511	NR_038366.1	
Forward primer		GCAAGGGGCGACTACTGTT
Reverse primer		AAGGGAGAGGCTAACTTGGC
miR-618	NR_030349.1	
Forward primer		GGTTGATGAGAGGAGGTGCT
Reverse primer		AGATTTTCCATGAGCTGCTGA
*MAEL*	NM_032858.2	
Forward primer		ACTATTTCTTCGTGCAGGAGAA
Reverse primer		GTTTCTGCTTCTCTGAGGGC
GAPDH	NM_002046.6	
Forward primer		CTCTCTGCTCCTCCTGTTCG
Reverse primer		TTGAGGTCAATGAAGGGGTC
U6	NR_004394.1	
Forward primer		ATTGGAACGATACAGAGAAGATT
Reverse primer		GGAACGCTTCACGAATTTG

### Microarray analysis

Microarray analysis was performed in KangChen Bio-tech (Shanghai, China). A LncRNA microarray was established using the GeneChip Human LncRNA Array 4.0 (Aksomics, Arraystar, Shanghai, China) in accordance with the manufacturer’s instructions. Significant Analysis of Microarray (SAM) software were used to analyze OS tissues and associated adjacent non-tumorous tissues samples (n=10) for differences in the expression of lncRNAs. First, double-strand cDNA (ds-cDNA) was synthesized from 5 μg of total RNA extracted from tissues using a SuperScript ds-cDNA synthesis kit. Then, the supernatant ds-cDNA was incubated with 4 μg of RNase A at 37°C for 10 min and cleaned using phenol. The purified cDNA was quantified using a NanoDrop ND-1000 (Thermo Fisher Scientific, Waltham, MA, USA) and labeled with Cy3. Then, 4 μg of Cy3 labeled ds-cDNA was used for hybridization, which was completed in Nimblegen hybridization buffer/hybridization component a in a hybridization chamber for 16–20 h at 42°C. Washing was performed in an ozone-free environment afterward with a Nimblegen Wash Buffer kit (Roche, Basel, Switzerland). Finally, the slides were scanned under an Axon GenePix 4000B microarray scanner. The differentialy expressed lncRNAs were selected according to the criterion of *P* < 0.05 and fold change value > 2.

### Cell transfection

LINC00511-targeting siRNA1 and siRNA2 (siRNA1-LINC00511 and siRNA2-LINC00511), *MAEL*-targeting siRNA (siRNA-*MAEL*) and target-free siRNA (si-NC), pcDNA3.1-LINC00511 (p-LINC00511), miR-618 mimics, and miR-618 inhibitor were purchased from GenePharma (Shanghai, China). The siRNA sequences are listed in Table 2. For transient transfections, HOS cells were seeded and cultured in 6-well plates. Then, the siRNAs, vectors, and miRNA mimics or inhibitors were transfected into 50% confluent cells using Lipofectamine 2000 reagent (Invitrogen). The medium was then replaced with fresh complete medium containing 10% FBS after 6 h of transfection.

**Table 2:** siRNA sequences of LINC00511 and MAEL.

**Gene**		**Sequence**
siRNA1-LINC00511	Sense	5′-GGAAGGUAGGGAGCAAACCUAUGAA-3′
	Antisense	5′-UUCAUAGGUUUGCUCCCUACCUUCC-3′
siNC-1	Sense	5′-GGAGUAGGGAGCAAACCUAUAGGAA-3′
	Antisense	5′-UUCCUAUAGGUUUGCUCCCUACUCC-3′
siRNA2-LINC00511	Sense	5′-CACAUGUUGCUUACAUGCUGCGUUU-3′
	Antisense	5′-AAACGCAGCAUGUAAGCAACAUGUG-3′
siNC-2	Sense	5′-CACGUUGAUUCGUACCGUCGUAUUU-3′
	Antisense	5′-AAAUACGACGGUACGAAUCAACGUG-3′
SiRNA-MAEL	Sense	5′-CCAGAUAUGUCAGCUUUGUCUUUAA-3′
	Antisense	5′-UUAAAGACAAAGCUGACAUAUCUGG-3′
siNC-3	Sense	5′-CCAUAUCUGCGAUUUCUGUUGAUAA-3′
	Antisense	5′-UUAUCAACAGAAAUCGCAGAUAUGG-3′

### CCK-8 and colony formation assays

Transfected cells (1×10^4^/well) were reseeded in 96-well plates and allowed to stand for 12 h at 37°C with 5% CO_2_. Then, CCK-8 reagent (MedChem Express, Monmouth Junction, NJ, USA) was added according to a standard protocol, and the absorbance value at 450 nm was measured by ELISA Elx800 (Bio-Tex, Winooski, VT, USA) every 24 h. For the colony formation assay, cells were resuspended in DMEM containing 10% FBS and cultured in 6-well plates (approximately 200 cells per well) at 37°C for 14 days. In particular, an additional round of cell transfection was performed when the cells were cultured for 7 days for the colony assays. After incubation, the medium was removed, and the cells were fixed with 70% ethanol and subsequently washed with ice-cold PBS. Finally, the cells were stained with a 0.1% crystal violet solution (Beyotime, Shanghai, China), and the number of colonies that contained fewer than 50 cells was counted under a light microscope (Olympus, Tokyo, Japan).

### Transwell assay

The migration and invasion capacities of transfected cells were detected by transwell assay. Migration assays began with seeded cells (6 × 10^4^ cells) that were transfected for 24 h in an upper transwell chamber, which contained serum-free medium (Corning, NY, USA). Serum-free medium was pre-laid by polyethylene terephthalate membrane (6.5 mm diameter, 8 μm pore size). The bottom chamber was filled with 10% FBS complete medium as a chemoattractant. After culturing in an incubator at 37°C for 24 h, the upper chamber and the membrane were disposed of, and the cells in the bottom chamber were fixed with 10% formalin and stained with a 0.1% crystal violet solution. For the invasion assay, a pre-laid polyethylene terephthalate membrane was coated with Matrigel (BD Biosciences, Bedford, MA, USA) before cell seeding. The cells on the bottom of the membrane were counted from five different microscopic fields and the average number was calculated.

### Lentivirus-mediated shRNA targeting LINC00511

shRNA-mediated knockdown of LINC00511 (lenti-sh-LINC00511) was performed using MISSION^®^ shRNA lentiviral particles (Sigma-Aldrich, St. Louis, MO, USA), which were designed to suppress the production of LINC00511 in HOS cells. The cultures that were transfected with shRNA lentiviral particles with nontargeting scrambled shRNA sequences were used as the control group (lenti-sh-ctrl). The top strand primer of sh-LINC00511 was 5′-CACCGGAGAAATAAGCTGG TGATTTCGAAAAATCACCAGCTTATTTCTCC-3′. The bottom strand primer of sh-LINC00511 was 5′-AAAAGG AGAAATAAGCTGGTGATTTTTCGAAATCACCAGCTTATTTCTCC-3′. HOS cells at a density of 3 × 10^5^ cells per dish were seeded onto 35 mm dishes. After 1 day of seeding, 200 μL of lentiviral particles in 2 mL of MEM-EBSS medium containing 10% FBS was added to the cultures, which were then incubated for 24 hours in 5% CO_2_ at 37°C. The cells that were successfully infected with lentiviral particles (lenti-sh-ctrl or lenti-sh-LINC00511) were selected using 3 μg/mL puromycin in the presence of 10% FBS for 48 hours. The cells were then harvested and subjected to qRT-PCR analysis to determine the efficiency of the lentiviral particles for suppressing LINC00511 gene expression.

### Xenograft mouse assay *in vivo*

Pathogen-free conditions were maintained for the lifespans of twenty male BALB/c nude mice (4 weeks old). Approval for the xenograft *in vivo* assay was obtained from Taizhou People’s Hospital. First, serum-free cell suspensions of untreated HOS cells (1×10^7^) were injected subcutaneously into the posterior flanks of nude mice; then, the nude mice were fed for 7 days. Mice were divided into 2 groups: (1) siRNA1-LINC00511; (2) NC (control siRNA). SiRNA1-LINC00511 or NC (control siRNA) was injected intratumorally twice a week. Besides, HOS stable cell lines with inducible shRNA targeting LINC005511 or shRNA control was established to repeat this experiment. Lenti-sh-LINC00511-transfected HOS cells (1×10^7^) or lenti-sh-ctrl-transfected HOS cells were injected into the tumors of the nude mice. Calipers were used to measure the tumor volumes, which were then calculated according to the length×width^2^/2 formula. The average tumor volume was measured 3 times every 3 days. At the termination of the experiment (the 21s day), the mice were sacrificed and the tumors were excised from each mouse to measure the average volume and weight.

### Dual-luciferase reporter assay

The miRNA target of LINC00511 was predicted using the miRcode v11 database, and miR-618 was selected as a promising candidate. Then, the mRNA target of miR-618 was predicted using the TargetScanHuman v7.2 database, and *MAEL* was selected. To test the target relationship between LINC00511 and miR-618, as well as between miR-618 and *MAEL*, a dual-luciferase reporter assay was carried out. First, the 3′-UTRs of LINC00511 and *MAEL*, containing the wild-type sequence (with a specific binding site for miR-618) or mutated sequence (without a binding site for miR-618), were cloned into a luciferase reporter vector (GeneChem, Shanghai China). HEK293T cells were co-transfected with a luciferase reporter plasmid, miR-618 mimics, and oligonucleotides (NC) for 48 h. Finally, the cells were harvested and subjected to a dual-luciferase reporter assay (Promega, Madison, WI, USA) to evaluate the Firefly and Renilla luciferase activities.

### Western blot analysis

Expression of *MAEL* was also detected at the protein level using Western blotting. In brief, total protein was extracted using a ProteoPrep^®^ total extraction sample kit (Sigma-Aldrich); 80 μg of protein extract was separated by SDS-PAGE and then electrophoretically transferred onto nitrocellulose (NC) membranes. Following blocking with 5% skim milk for 1 h, the membranes were incubated with rabbit anti-*MAEL* (1:1000 dilution, Santa Cruz, CA, USA) at 4°C overnight. After washing three times with PBST, the membranes were incubated with HRP-labeled goat anti-rabbit IgG (1:500 dilution, Santa Cruz) for another 1 h. Additionally, GAPDH was applied as an endogenous control. Protein bands were visualized with an ECL detection kit (Amersham Pharmacia Biotech, NJ, USA) and quantified using ImageJ software 1.8 (National Institutes of Health, Bethesda, USA).

### Statistical analysis

All data were generated from triplicate independent experiments and are presented as the mean ± SD. Statistical calculations were carried out with GraphPad. Two-tailed Student’s t-test or Chi-square test was performed to calculate the differences between two groups, and statistical significance was set as *P* < 0.05.

### Ethics approval

All procedures performed in studies involving human participants and animals were in accordance with the ethical standards of The Taizhou People’s Hospital. Informed consent to participate in the study has been obtained from participants.

## Supplementary Material

Supplementary Figure 1
