# Supplementary material for: Long intergenic non-protein coding RNA 511 promotes the progression of osteosarcoma cells through sponging microRNA 618 to upregulate the expression of maelstrom
Source: Aging (Albany NY). 2019 Aug 6;11(15):5351–67. doi: 10.18632/aging.102109 (PMC6710040; doi:10.18632/aging.102109)
Supplement: Supplementary Figure 1 [file aging-11-102109-s001.pdf]

SUPPLEMENTARY FIGURE

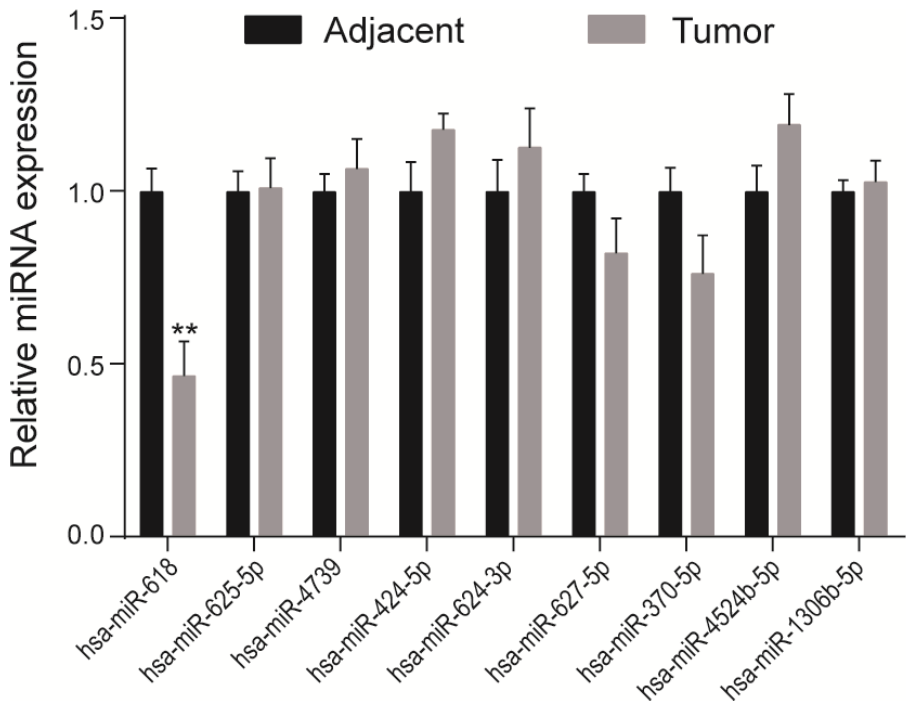

Supplementary Figure 1. MiRNA expression levels of 9 miRNAs, including miR-618, in OS tissues and normal tissues. Nine miRNAs expression levels were detected in OS tissues and adjacent normal tissues by qRT-PCR assay. The miR-618 expression level in OS tumor tissues was lowest among the 9 miRNAs. ***P*<0.01 compared to the adjacent group.
